# Gynæcology

**Published:** 1880-11

**Authors:** 


### GYNÆCOLOGY.

ON INFLAMMATION OF THE VULVO-VAGINAL GLAND.—Martineau. (*France Médicale*, July 21, 1880).

The vulvo-vaginal glands or glands of Bartholin, two in number, are found on each side of the vulva, in a triangular space bounded by the vagina within, the rectum behind and the ascending branch of the ischium without; they correspond to the inferior segment of the labium majus and their excretory duct opens without the hymen or its débris, the carunculæ myrtiformes in the furrow which separates them from the labia minora at the junction of the lower fourth with the upper three-fourths of the vulvar ring.

This acinous gland may become inflamed, as well as its excretory duct, under the influence of the causes we are about to study, and there results therefrom sometimes a painful infirmity to the woman on account of the persistence of fistulæ. The history of this inflammation has been traced with a master's hand by Huguier, who had but one fault, that of dividing his subject without measure, of distinguishing, notably, stages which are only degrees of the inflammation. However, his description has never been surpassed, and, following the example of my predecessors, I shall frequently borrow from him.

This affection is especially observed between 17 and 3[illegible] years; it is very rare before puberty, and it is equally unfrequent in old women. Among 37 cases which have presented themselves during my service during a space of three years, I have found that 35 were between 16 and 23 years, one of 30 and one of 42 years. It is then, as seen, an affection belonging to the genital and sexual period of women. It shows itself when the physiological functions appear. The causes therefore, are generally sexual excesses, disproportion of the genital organs, menstruation, contagious affections, such as gonorrhœa of the vulva or vagina, traumatic injuries of the genital organs, rape, difficult defloration, repeated masturbation, saphism and friction of the vulva by the use of sewing machines.

Thus in the 37 cases of my service about a dozen cases were caused by abuse of coitus; I have observed several in my practice, and there is no physician who has not had an opportunity of seeing it in his private practice.

Among all these cases I will cite the two following:

The first was the case of a young woman of 22, who, meeting her lover, from whom she had been separated during the siege of Paris during the commune in 1870–71, submitted to his embraces nine times in one night. There followed from it an abscess of the left vulvo-vaginal gland, for which she sought my advice. The cure was complete at the end of eight days.

The second case was that of a young woman of 25 who lived with her parents and only saw her lover evenings. One evening between the hours of 8 and 11 coitus was repeated seven times. Two days after there was slight itching of the labium majus, then smarting and swelling; the patient entered the hospital and I found an abscess of left vulva-vaginal gland, which healed at the end of eight days.

These are not isolated cases. The gynæcologists mention several and I doubt not physicians have occasion to see them in their practice, especially in young brides, where to excessive coitus must be added, difficulty of defloration and the fatigue of the bridal trip. A. Tardieu observed a case as the result of a rape; he cites a case in a girl of sixteen who was obliged to submit to the repeated assaults of her ravisher at least twenty times in less than eight days.

Various explanations have been given of the pathogenesis of this affection. Huguier thinks that in these cases the inflammation of the gland is the consequence of the functional superactivity of the gland excited by the voluptuous sensations of the sexual act; also, he admits that it is produced without this act, as by the act of masturbation, and the reading of erotic books. At first, he says, there is only a hypersecretion of the glandular fluid, then the fluid becomes cloudy, and soon after clearly purulent; the gland, which is functionally unusually active, swells and inflames. Other authors think that the immediate cause is the friction of the male organ against the gland. It is to this cause, in my opinion, that we must attribute the inflammation of the gland. Indeed there is an attrition, a traumatic injury, and this is all the more intense where there is a disproportion of the organs. It is notably to this cause that we must attribute the inflammation of the gland which comes on after the first sexual act, when the connection is difficult and painful. The following case is a striking example.

In June, 1877, a young girl of seventeen years entered ward Saint Alexis for an abscess of the left vulvo vaginal gland. A first coitus had taken place six months before, but it had been incomplete and very painful; a second attempt, followed by success, but at the price of great suffering, had been tried eight days before the patient entered the hospital. Now, on being interrogated about the defloration, she reports that the penis of her lover was enormous. The cure took place eight days after, by the spontaneous opening of the abscess.

A fact which corroborates this opinion is that the glandular inflammation is seated especially on the side where the friction of the penis is most felt. Is it an effect of the position taken by the woman or by the man? Is it from some other cause? It is always the case that most of my patients complain spontaneously of this sensation of friction being stronger on one side than the other. I will add that the abscess is ordinarily produced on the left side; now it is, in fact, on this side that the friction of the penis is the most acutely felt during the sexual act. Is it because it corresponds to the right side of the man who has an exaggerated tendency to lean to this side? I do not know. I limit myself to stating this fact for your meditation, only insisting upon the fact of development of the abscess upon the side where there is the most friction. I will add that in most of my patients the voluptuous spasm invoked by Huguier as the cause of the inflammation, was absolutely wanting.

In the cases which recognize masturbation for their cause, it is still repeated friction with the hand, with the penis, the attrition produced by pressure of the thighs, the traumatism consequently, much more than the voluptuous excitation admitted by Huguier. The traumatism of manualization is notably evident in the following case:

It is the case of a young woman of twenty-one years, who entered Ward St. Alexis in October, 1879, for an abscess of the left vulvo-vaginal gland developed in consequence of handling on several occasions by her lover. This patient was aware that the friction was not solely of the clitoris, but also of the left side of the vulva. Moreover it is clearly to a traumatic cause that this glandular inflammation must be attributed, for in girls before puberty, in whom the vulvo-vaginal gland has not yet attained its developments, it is not seen. It is even rarely observed in adult women who are addicted to excessive masturbation. Thus, among our patients who so often present those characteristic deformities of masturbation, which I have taught you to recognize, abscess is relatively rare, notwithstanding the frequence of voluptuous sensations. I reject then the explanation of Huguier and accept the more direct influence of traumatism. At the most the repeated functional excitement of the gland might constitute a predisposition. I reject equally the opinion of M. A. Guérin, who thinks that coitus frequently repeated without reaching the final spasm produces a hypersecretion and a hypertrophy of the gland which may end in inflammation. Although I carefully question my patients on this point, which interests me specially for the pathogenisis of constitutional metritis, I have never found the slightest connection between incomplete coitus and inflammation of the vulvo-vaginal gland. One traumatic cause, in some sort professional, of inflammation of the gland of Bartholin, is the use of the sewing machine. It was the cause in the case of a young woman of twenty-four, who entered September, 1877, for an abscess of the left vulvo-vaginal gland, occurring after ten hours' continuous use of the sewing machine daily for a week.

By the side of these traumatic cases is ranged an order of causes of which the etiologic importance is considerable. I speak of vulvo-vaginal gonorrhœa. Of the 37 cases in my service, in 25 the inflammation was due to gonorrhœa. It is generally located on one side only, being on both sides in only two cases. In fact, gonorrhœa has a predilection quite peculiar for the vulvo-vaginal gland; it settles and in some sort remains there. It is well, gentlemen, to be forewarned of this seat of gonorrhœa in women, for not only may it escape detection and become a cause of contagion, but it is the source of frequent recurrences by auto-inoculation. This localization of the specific inflammation explains the important rôle which it plays in development of abscess of the vulvo-vaginal gland, at the same time that it explains the fact, which is indeed not common, of abscess in both of these glands.

Simple vulvitis sometimes gives rise to this glandular inflammation. It is especially the case when it is of a constitutional nature. Otherwise it is only exceptionally that it gives rise to it; a traumatic cause may always be suspected.

In terminating this etiologic study, I should call your attention a moment to the relations which exist between the development of inflammation of the vulvo-vaginal gland and menstruation. Thus, in three of my patients, the inflammation was developed during the menstrual period.

The first of these patients had had twenty abscesses of the vulvo-vaginal gland, all coming on at the menstrual period. The second had frequently had abscesses at the menstrual period, but was uncertain as to the number, while the third entered with an abscess for the first time at the menstrual period. It appears then that the courses constitute a predisposition to abscess or at least favor recurrences of an inflammation imperfectly cured.

You have without doubt, gentlemen, been struck by the fact that the abscess is seated more frequently on the left than on the right. Thus in our thirty-seven cases twenty-three were on the left side, twelve on the right and two on both sides at the same time. Why this predilection for the left side? I avow that in the presen state of the science, apart perhaps from certain traumatic cases, I cannot give an explanation applicable to all cases.

Let us pass now to the study of the symptoms. At first slight heat is felt, mild pruritus and moisture of the vulva. If the glands are palpated, one is felt to be more prominent than the other; at the same time the palpation provokes a slight pain and reveals a little stringy mucus which has already become somewhat cloudy by the presence of pus globules. The next day and the following days these symptoms increase; the pain becomes very sharp, examination locally reveals between the small lip, which is pushed inward, and the greater lip, which is pushed outward, a small hot tumor, tense and painful, which may acquire the size of a small pear, and which obliterates the vulvar orifice within. This tumor is pear-shaped; it is more prominent inward than outward, more below than above, and corresponds with the inferior segment of the greater lip; of which the upper portion undergoes no change. The corresponding portion of the smaller lip is tense and smooth, as well as the carunculæ myrtiformes. By palpation we recognize that this tumor is painful, rounded, limited, more or less resisting or fluctuating, according to the degree of inflammation. The mucous membrane is red, hyperæmic, sometimes violaceous, shining and tense over the tumor.

At the end of some days, fluctuation becomes clearly marked and the abscess opens spontaneously, to the great comfort of the patient, generally upon the mucous membrane in the vicinity of the orifice of the excretory canal or in the canal itself, from which the pus is seen to flow upon pressure. The pus is creamy, inodorous, sometimes mixed with blood. When there is at the same time purulent gonorrhœal vaginitis, the pus exhales sometimes a fetid, nauseous odor. There occurs then what is seen in purulent collections, developed in the vicinity of natural cavities, such as the intestinal tube—the pus takes by a sort of osmosis the putrid odor of the gases and matters contained in these cavities; the same here, the pus of the abscess borrows from that of the vagina its special nauseous odor.

The evolution of the inflammation of the vulvo-vaginal gland is rapid: in eight or ten days the cure is generally complete. Induration of the glandular tissue often remains; the abscess then re-forms, especially at the menstrual epoch; M. Gallard has seen several examples of it. In other cases, the opening remains fistulous, and this constant purulent discharge becomes a cause of hypochondria for the woman, and a port of entry for gonorrhœa and syphilis. The prognosis is not then absolutely favorable, and there may be occasion to resort to a surgical interference.

The diagnosis is generally easy. This pyriform tumor, situated at the entrance of the vulvar ring, between the labium majus, which it pushes outward, and the labium minus, which it flattens within, clearly detached from the neighboring parts and leaving intact the superior segment of the vulva, cannot be confounded with a stercoral abscess, with a presecto-vulvar abscess, nor with a purulent collection, proceeding from caries of the ischium. One will not confound this abscess with abscess of the labium majus. In fact, phlegmon of the greater lip is seated at the external portion of the vulva; it projects outward, not inward, like abscess of the vulvo-vaginal gland; finally, while the latter is habitually circumscribed and unilateral, phlegmon often becomes general, extending to the labia minora, the clitoris, and even, according to Huguier, to the mons veneris.

It is more difficult to diagnose between abscess and a cyst of the excretory duct. In both cases we find, on one side of the vulva, a globular tumor of limited extent, springing from the interior of the vulvar ring, and pushing the large lip outward; but in case of cyst, it is smaller, indolent, without reactionary and inflammatory phenomena; its greater diameter is directed according to the transverse direction of the excretory duct, and pressure causes the escape of a colorless fluid, slightly viscous but not purulent.

The pathogenic diagnosis is important, because it enables the physician to take measures to prevent a return of the affection.

The therapeutics of inflammation of the vulvo-vaginal gland is very simple. At the beginning, emollients, poultices of fecula, bran or starch baths. Flax-seed meal should not be used, as it becomes rancid, and irritates the vulvar mucous membrane. The patient should be kept in bed. When the abscess is formed, should we await its spontaneous opening or use the bistoury? M. A. Guérin has studied this question, and I fully agree with his conclusions. Fistulas are much more common when one hastens to open the abscess; it is much better to wait until it opens spontaneously through the mucous membrane or through the excretory canal. It generally opens in the nympho-labial furrow, and the cure is speedy. This is the plan I generally follow. I advise you, then, not to interfere, especially if the abscess is due to gonorrhœa. Still, when the pain is too severe, you may operate to relieve the suffering of the patient. In this case use the lancet or bistoury, and open the abscess in the nympho-labial furrow, near the orifice of the excretory duct of the gland. M. Gallard has recourse to a large incision, which he prolongs to the fourchette, in order to avoid the formation of purulent foci. In all cases it is best to place a piece of charpie in the wound to prevent too rapid union. If there exists a tendency to formation of a fistula, it is best to cauterize the surface of the focus with the crayon of nitrate of silver. In certain cases this practice is insufficient; the abscess is constantly reproduced, and becomes a real infirmity; it is then necessary to resort to excision of the gland. For this an incision is made in the nympho-labial fold, then separate the lips of the wound and cautiously dissect out the gland; when it is well separated from the surrounding parts, remove with the bistoury or scissors. The arteries should be carefully ligated, or they may bleed profusely, according to M. A. Guerin. As a dressing, a piece of charpie, saturated with a one per cent. solution of chloral, or a one-tenth per cent. solution of carbolic acid, should be laid over the wound.

SIMPLE AND FOLLICULAR VULVITIS.—(M. Gallard, *Journ. de Méd.*, p. 355, August, 1880.)

In a clinical lecture at La Pitié, the author said that the inflammations of the vulva were of two different forms—simple vulvitis and follicular vulvitis. Simple vulvitis is encountered much more frequently in little girls than in women, due, probably, to the fact that the former are not so cleanly in their habits as the latter; moreover, there sometimes occurs in them a true catarrhal inflammation, connected in certain cases with dentition. But in the child a medico-legal question is often raised, and one is frequently called to decide whether a vulvitis is of traumatic or spontaneous origin. Here it is important to recall a preliminary fact; it is that up to the age of five or six years, the vulva is much more prominent than in women, which might easily cause one to believe swelling to be present, when in reality there is none. This cause of error being known, we may admit, in a general way, that in vulvitis of traumatic origin it is very rarely that one does not find upon the genital parts, between the labia majora, or the fourchette, or even on the internal surface of the thighs, traces of contusion, or abrasions of such a nature as to cause us to recognize the wounds and injuries committed. In general, also, traumatic vulvitis is more circumscribed, confined to the vulva or meatus, and unequally distributed upon the parts affected. As to whether the discharge is simple or virulent, it is a question almost impossible to answer upon a first examination; it is only by the rapid course of the malady, which disappears rapidly if it be of simple traumatic origin, but which, on the contrary, persists a longer time if it is at the same time of gonorrhœal origin, that one can be positive on this subject. All these inflammations, however, whether they depend upon uncleanliness, fatigue or a true traumatism, disappear ordinarily very quickly under the influence of rest, emollients and appropriate baths, and these are the only means necessary for their cure.

By the side of this general inflammation is to be placed follicular vulvitis, in which the inflammation is localized, sometimes in the sebaceous glands, sometimes in the hair follicles. At the beginning, it is only manifested by slight elevations situated, principally, upon the labia majora, accompanied by swelling and intense itching, then these small elevations rapidly increase in size and are transformed into small ulcerations which may either cicatrize very rapidly or remain ulcerated for a long time. It is at this period especially that diagnosis presents difficulties, but there is one important fact to be remembered; it is that one may always, by the side of these ulcerations, encounter follicles in the first stage of induration, forming small pimples easily recognized. It is principally on this character that is based the diagnosis between herpes, as well as the soft and hard chancres. It may happen, also, that in the latter case inoculation of syphilis takes place in these numereus ulcerations, and there results a multiplicity of chancres entirely abnormal.

This affection also arises from accumulation of the products of secretion, from the excitement produced by menstruation, and sometimes by scratching provoked by the presence of pediculi. Hence all irritating means, such as toilet vinegars and cosmetics, should be proscribed, and either alkaline substances or emollients be used.

